# Understanding gender segregation through Call Data Records: An Estonian case study

**DOI:** 10.1371/journal.pone.0248212

**Published:** 2021-03-25

**Authors:** Rahul Goel, Rajesh Sharma, Anto Aasa

**Affiliations:** 1 Institute of Computer Science, University of Tartu, Tartu, Estonia; 2 Department of Geography, University of Tartu, Tartu, Estonia; Universitá degli Studi di Milano, ITALY

## Abstract

Understanding segregation plays a significant role in determining the development pathways of a country as it can help governmental and other concerned agencies to prepare better-targeted policies for the needed groups. However, inferring segregation through alternative data, apart from governmental surveys remains limited due to the non-availability of representative datasets. In this work, we utilize Call Data Records (CDR) provided by one of Estonia’s major telecom operators to research the complexities of social interaction and human behavior in order to explain gender segregation. We analyze the CDR with two objectives. First, we study gender segregation by exploring the social network interactions of the CDR. We find that the males are tightly linked which allows information to spread faster among males compared to females. Second, we perform the micro-analysis using various users’ characteristics such as age, language, and location. Our findings show that the prime working-age population (i.e., (24,54] years) is more segregated than others. We also find that the Estonian-speaking population (both males and females) are more likely to interact with other Estonian-speaking individuals of the same gender. Further to ensure the quality of this dataset, we compare the CDR data features with publicly available Estonian census datasets. We observe that the CDR dataset is indeed a good representative of the Estonian population, which indicates that the findings of this study reasonably reflect the reality of gender segregation in the Estonian Landscape.

## 1 Introduction

Segregation has long been assumed to play a critical role in many developing countries’ socio-economic structure and overall stability [1]. According to [2], consequences of segregation are not limited to developing countries, but the detrimental impact of segregation is more severe in countries with poor political and legal structures. As a result, a great deal of emphasis is required towards policies to facilitate integration and interaction in diverse societies.

In the past, the research on segregation has been constrained by a lack of credible data. As a consequence, many of the previous research work relies on conventional government census data [3]. However, census data can capture the precise pattern of the physical settlement but rarely record trends of social interaction, which are necessary to develop a thorough understanding of the essence of social interaction.

In this paper, we use the *Call Data Records* (CDR) to understand segregation in Estonian society. Over the last decade, CDR has been analysed from a number of perspectives, such as social network analysis [4], sociocultural aspects of a city [5], identifying the human mobility patterns [6], understanding calling patterns using phone call duration [7], impact of various events on calling activities [8] and population distribution [9] to name a few. lso, CDR often integrates with other data such as traffic data [10], financial data [11], and GIS data [12] for a deeper understanding of human behavior. Previous studies have also used CDR data to explain the ethnic discrimination in society using call duration [7, 13], and social group discrimination at the workplace [14]. This work investigates gender segregation within society by analyzing the users’ characteristics and their interaction through social network analysis.

In this work, we analyze anonymized CDR data provided by one of the leading mobile operators in Estonia to cover the following research directions:

- **Macro-analysis**: In this analysis, we explore the social network interactions to understand gender segregation using CDR. We further investigate and compare various properties of females-only and males-only networks separately. Additionally, to identify the relevant users in the network, we employ the PageRank centrality algorithm. The network is also explored to identify gender representation in various counties in Estonia (Section 4.1).
- **Micro-analysis**: During this analysis, we investigate users’ characteristics such as age, language, and location to understand gender segregation in detail. We also analyze the users’ interactions based on gender, age-groups, language, and locations (Section 4.2).

The findings of our social network analysis suggest that the males-only network relatively dense and firmly connected while the females-only network is spread out. Also, our analysis using users’ characteristics, such as gender, age, language, and location show that the prime working-age population (i.e., (24,54] years) is more segregated than other age groups. We also find that the Estonian-speaking population (both males and females) tend to interact more with other Estonian-speaking, specifically the same-gender individuals.

We also demonstrate that CDR data can be used to deduce the relationships between the user’s characteristics that are quite similar to actual relationships in society by comparing the CDR data with publicly accessible census datasets. Thus, CDR data can be used to understand segregation and can be helpful for government agencies to make target policies for needed groups.

The rest of the paper is organized as follows. Next, we discuss related works. We then describe the dataset in Section 3. Section 4 presents the results of our descriptive analysis of the dataset and we conclude with a discussion of future directions in Section 5.

## 2 Related work

In this section, we discuss works related to Call Data Records (CDR) and segregation at the intersection of which this work lies.

Over the last decade, CDR has attracted a lot of research. In [5] and [15], the authors showed that CDR data can provide valuable information regarding the social structure of societies when analyzed using social network analysis. The strong and weak ties between individuals in social networks are identified by authors in [16]. In another work, population density is calculated using CDR [9]. A fair amount of research has also been done using CDR data for identifying mobility patterns. For example, [6, 17] authors demonstrate that the human path is predictable and reproducible. In [18], the authors proposed a human mobility model and validated using a real dataset from New York and Los Angeles metropolitan areas. In [19], the authors analyzed human behaviour to find the movement pattern across various age-groups.

A set of works have also focused on understanding the varying types of segregation among society using CDR. Four types of factors appear to contribute to segregation are discrimination, disadvantage, preferences, and social networks [20, 21]. For example, authors in [13] studied the temporal variation of ethnic segregation in the city of Tallinn, the capital of Estonia. Their findings revealed that segregation is significantly lower on workdays and during the summer holidays. In a different work, segregation is decomposed into two types i) social segregation: observed in interactions among people, and ii) spatial segregation: determined by the physical locations of people [22]. Furthermore, a framework is proposed to model and measure fine-grained patterns of segregation from large-scale digital data.

In another line of work, authors studied the immigrants’ segregation in Estonian society [23] using census data and passive mobile positioning data (CDR). Their results showed that the activity space of Russian-speakers of all age-groups is smaller and less diverse than those of Estonians; and also revealed that there is higher ethnic segregation in younger age-groups. Discrimination and prejudice by the dominant group restrict the activities of the members of minority groups [24]. Even though discrimination is illegal in most countries, and the societal tolerance of minorities has increased, discrimination is still present in everyday life [25]. In another work [26], authors showed that segregation, isolation, and homophily can be measured by deriving population estimates from CDR. Their findings revealed that the evolution of refugees’ communication patterns and mobility traces can provide insights into their social integration.

Studies of segregation in workplaces have shown a concentration of minority groups in certain employment niches and workplaces [14]. It has been suggested that segregation in places of residence and segregation in places of work are connected. However, workplace-based segregation is lower than residence-based segregation [27, 28]. Outside of the place of residence and the place of work, ethnic differences have also been studied mainly through single measures of leisure activities, such as going to church [29], casinos [30], or national parks [31, 32].

This work is different from [7, 13] as the focus of their works was mostly on phone call duration to understand human behavior. However, in this work, we analyze CDR data to understand gender segregation using social network analysis and feature analysis based on gender, language, age, and location.

## 3 Dataset

The analysis utilizes the anonymized call data records (CDR) provided by a leading mobile operator in Estonia. The dataset includes timestamp data to the level of seconds for each call activity, and the passive mobile positioning of the cell phone tower. The call records span six days, that is, from May 8, 2017, to May 13, 2017. The data consists of 12,317,970 unique call records from 1,175,191 unique users which is 89.32% population of Estonia [33].

For each call activity, the following information is available: randomly generated user *pseudonymous ID*, *timestamp* (with an accuracy of 1 second), and *location* of the network cell. The pseudonymous ID guarantees the user’s privacy, which cannot be connected to a specific individual or phone number. Additionally, for research purposes, the *gender* of the user, *year of birth*, and preferred language of communication is provided. The options for the preferred language of communication are either *Estonian*, *Russian*, or *English*, as chosen by the user when signing the contract with the service provider. Please note that not all users have additional (gender, language and location) information in the dataset. For example, *gender* information is available for 130,988 users in which 61,933 are males and 69,055 are females. Table 1 summarises various statistics about this dataset.

**Table 1:** Statistics about the dataset. Users’ gender count are listed individually under various features to provide a comprehensive insight into the dataset. For example, under feature *Languages*, users are categorized in three languages that is *Estonian*, *Russian*, and *English*. The number of males and females under each category is also listed.

Parameters	Value
Time period	May 8, 2017 to May 13, 2017
Call Records	12,179,970
Unique Users	1,175,919
No. of Features	37
Gender; #Male/Female	61,933/69,055
**Age-Groups**	
(0,14]; #Male/Female	5/4
(14,24]; #Male/Female	99/59
(24,54]; #Male/Female	30,238/33,864
(54,64]; #Male/Female	12,012/14,082
(64,100]; #Male/Female	9,027/14,327
**Languages**	
Estonian; #Male/Female	45,888/54,823
Russian; #Male/Female	7,072/7,629
English; #Male/Female	142/66

Based on the official age-group categorization proposed by *Europe-Bureau* and *Statistics Estonia* [34, 35], we categorise users’ age into the following five categories:

1. 0-14 years: Children.
2. 15-24 years: Early working age.
3. 25-54 years: Prime working age.
4. 55-64 years: Mature working age.
5. 65+: Elderly.

In Table 1, the *Age-Groups* row provides the distribution of the users in the dataset according to *gender* and *age-group*. E.g., for the age-group *(24,54]; #Male/Female* with value: *30,238/33,864* means that for age-group (24,54], 30,238 users are males and 33,864 users are females.

**Fig 1:**
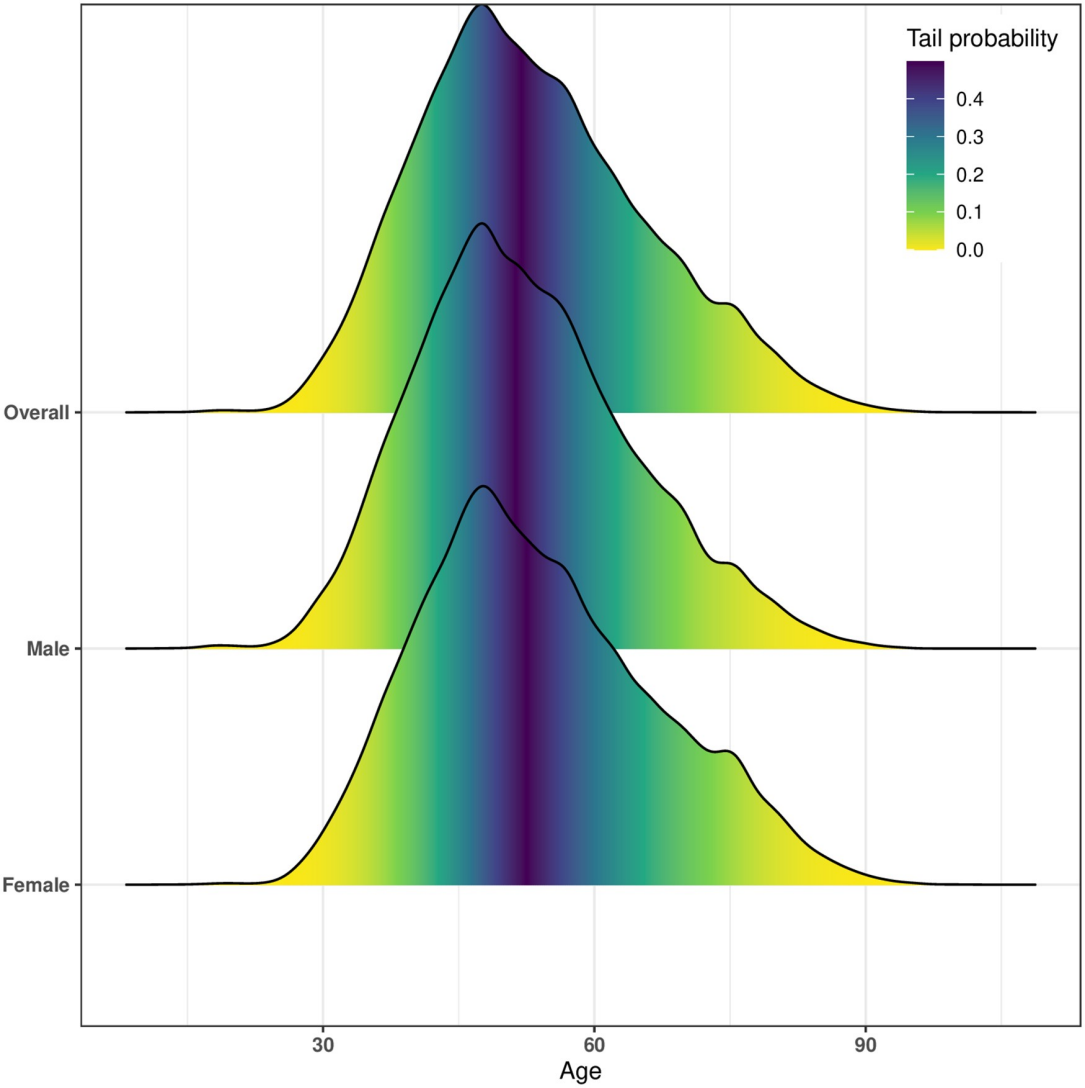
Calls density with probabilities based on age. X-axis represents the user’s age and y-axis represents the call density for *Overall*, *Female* and *Male* users in CDR data. Using the cumulative density function for the distribution, we map the tail probability directly into colors. For example, the 25^th^, 50^th^ and 75^th^ quantile for overall users is 44, 52 and 61 respectively. Similarly, these quantiles for female users are 44, 52, 62; and for male users are 44, 51, 60.

The age distribution based on call density for overall users (both females and males), females-only users and males-only users is shown in Fig 1. Please note that we use the word *“user(s)”* in case of CDR data individuals, but *“population”* while referring to the actual population of Estonia. The median age for overall, females-only and males-only users are 52, 52, and 51 years respectively. It is to be noted that in 2017, the median age of the population in Estonia was reported as 41.6 years (https://www.statista.com). Since the use of mobile phones is prevalent after a certain age, the difference between the median age of the actual population and from CDR users is apparent. Please note that due to only 9 total users in the age-group (0,14], we have excluded this age-group for further analysis.

## 4 Descriptive analysis

In this section, we first present the findings of our **macro-analysis** to understanding gender segregation, performed by exploring the social network interactions of the CDR (Section 4.1). Next, to explore the segregation in detail, we discuss the results of our **micro-analysis** considering various users’ characteristics such as age, language, location, etc (Section 4.2).

### 4.1 Macro-analysis

**Fig 2:**
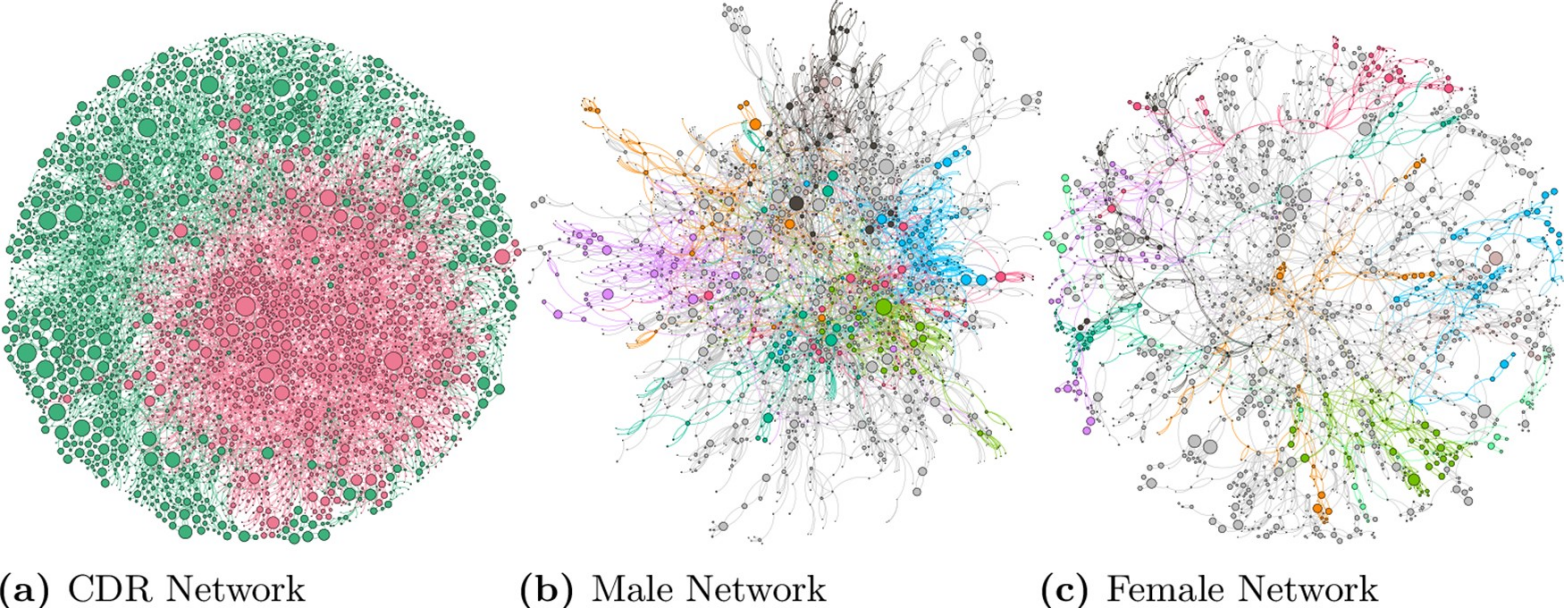
Users network formed using CDR data. A representative of the original network using snowball sampling. In Fig (a), users are color-coded based on *gender*. Color coding is as follows: red node are male users and green nodes are female users. The nodes with higher PageRank value are shown in relatively bigger size than others. Fig (b) shows the *males-only* network (with modularity = 0.803). Color-coded group represents communities. Similarly, Fig (c) shows the *female-only* network (with modularity = 0.913). Here, also color-coded group represents communities.

We create a directed network that represents the call connections among users where an edge (*u* → *v*) is formed if a user *u* has called user *v*. Fig 2(a) shows the CDR network, where each node is color-coded based on the gender. The red nodes represent male users, and green nodes represent female users. Links between users are also color-coded. Links which originate from males are colored red (i.e., calls from male to male; and male to a female), and similarly links that originate from females are colored green (i.e., calls from female to female; and female to male).

Furthermore, we employ the well-known *PageRank* algorithm [36] to identify the centrality of nodes in the network. *PageRank* reflects the importance of a node in terms of its influence in the network. For example, an individual with a higher Pagerank could reflect its bigger social influence in propagating a piece of information in the network. In Fig 2(a), the size of the node reflects the Pagerank of the node.

Table 2 provides the statistic of the network. The lower value of the average clustering coefficient and edge density can be used to infer that the network is sparse. The values of these metrics further indicate that the network is spread out and the transmission of information would possibly take longer to transmit throughout the network. From the values of strongly and weakly connected components size and the number of components, we can conclude that there are a large number of small communities. The value of reciprocity indicates that only 24.6% individuals have mutual interests with each other.

**Table 2:** Network statistics of users.

Network Properties	Value
Nodes	1,175,919
Edges	4,664,821
Average in-degree	3.97
Edge density	3.37e-6
Number of triangles	2,909,058
Average clustering coefficient	0.096
Strongly & weakly connected component size	1,170,309
Number of components	14870
Reciprocity	0.246

Based on color-coding, we can easily detect clusters of males and females in the CDR network (Fig 2(a)). For further studying the segregation, we study the males-only (see Fig 2(b)) and the females-only (see Fig 2(c)) networks separately. For creating the males-only network, we drop all the caller and callee ids, which belong to females. Similarly, we drop all the caller and callee ids which are males for creating the females-only network. For better comparisons of these networks, we report the properties of each of these networks in Table 3. Although this creation of males-only and females-only networks is synthetic, nevertheless it can provide some significant information about segregation in these networks.

**Table 3:** Statistics comparison of male and female network.

	Male	Female
Nodes	40,711	45,931
Edges	76,188	64,824
Edge density	4.6e-5	2.9e-5
Average clustering coefficient	0.097	0.138
Average path length	10.46	24.65
Diameter	136	203
Modularity	0.803	0.913

In Fig 2(b) and 2(c) users are grouped into communities based on modularity values (0.803 for the males-only and 0.913 for females-only network). The higher value of modularity indicates that the females-only network has more clusters but these clusters are densely connected within themselves as also supported by the higher average clustering coefficient of the females-only network, which suggests that females bonds in smaller groups, but these groups are tightly connected compared to their males counterparts. Higher value of edge density for males-only network suggests that males, in general, have more connection compared to females. In addition, smaller diameter and average path length values of the males-only network indicate that the network is compact compare to the females-only network, which is more spread out. These males-only and females-only network metrics point out that the transmission of information is fast in the males-only network compared to the females-only network.

**Fig 3:**
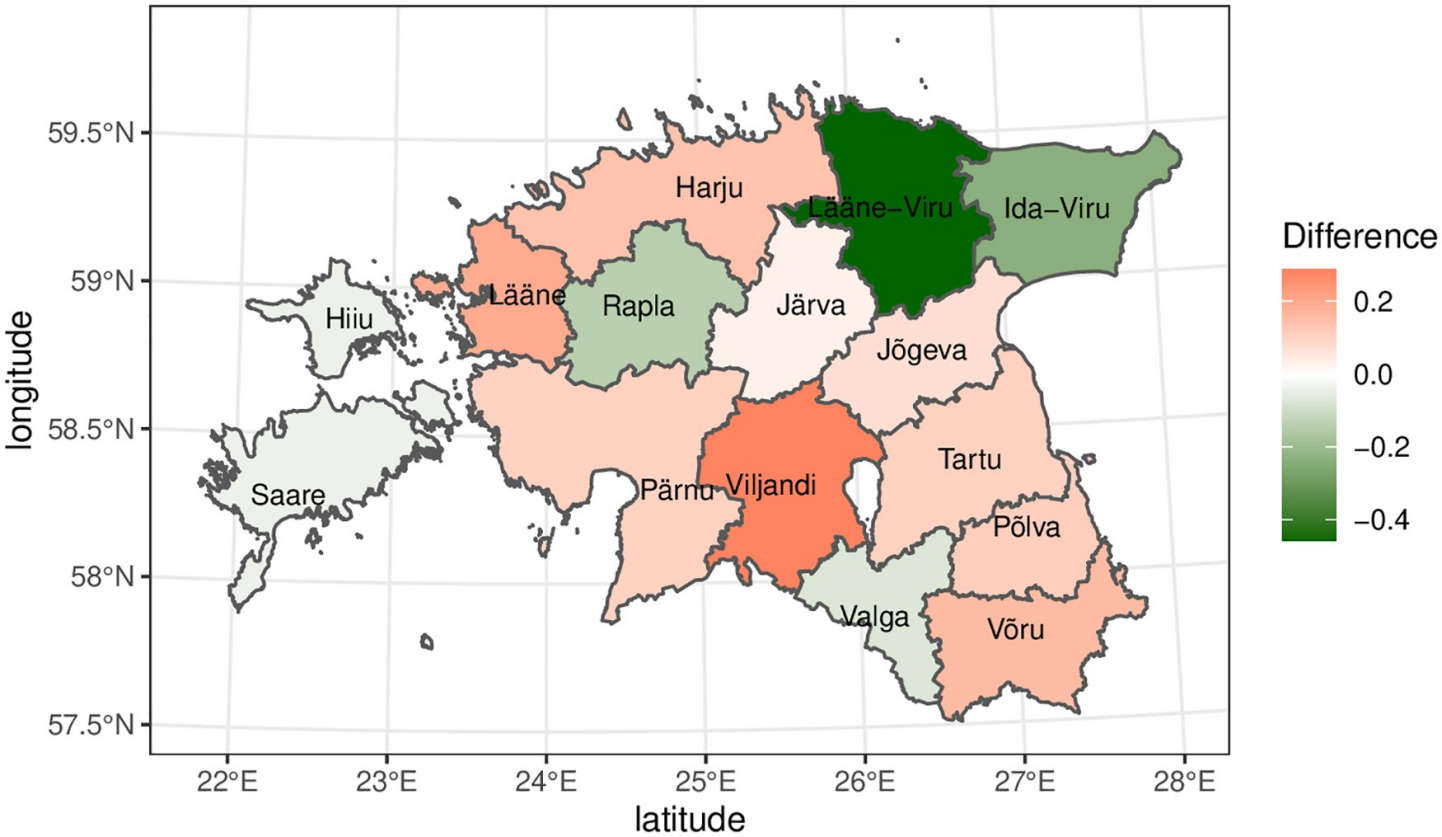
The difference between PageRank gender representation ratio and actual population gender distribution ratio among various counties. The difference less than zero (in green) indicates that females are major source of information in that region and their number is higher compared to their population. Similarly, the difference greater than zero (in red) implies that in that region, higher number of males are the primary source of information compared to their population.

With the aim to understand the gender predominance in different counties of Estonia, we further looked at the top-100 influential users in the CDR network by using the PageRank centrality. In Estonia, there are 15 counties, with Harju county, which includes capital Tallinn being the most populous and Hiiu county being the least populous. The gender distribution of the top identified nodes is shown in Table 4 (column 2). The actual gender population distribution among counties is shown in Table 4 (column 3). The difference is calculated by subtracting actual population ratio from the PageRank centrality ratio. From the difference (see Table 4, Column 4), we can conclude that in six counties (Lääne-Viru, Ida-Viru, Rapla, Valga, Hiiu, and Saare), there are more female influencers among top-100 nodes taking in account these counties population. We can conclude from the difference (see Table 4, Column 4) that there are more female influencers among the top-100 nodes in six counties (Lääne-Viru, Ida-Viru, Rapla, Valga, Hiiu, and Saare), taking into account the population of these counties. Similarly, there are more male influencers in total of 11 counties such as Viljandi, Lääne, Võru, etc (see also Fig 3) [37].

**Table 4:** Comparison of population gender distribution and PageRank centrality gender distribution (among top 100). Difference is calculated by subtracting population ratio from PageRank ratio. The negative value of difference indicate that the female representation is higher in that county. Similarly, a positive difference value indicate that male representation is higher in that county.

County	PageRank centrality (% Males/Females)	Population dist. (% Males/Females)	Diff
Harju	49/51	45/55	0.1426025
Hiiu	47/53	48/52	-0.03628447
Ida-Viru	37/63	45/55	-0.2308802
Jõgeva	50/50	48/52	0.07692308
Järva	48/52	47/53	0.03628447
Lääne	52/48	47/53	0.1965409
Lääne-Viru	30/70	47/53	-0.458221
Põlva	51/49	48/52	0.1177394
Pärnu	49/51	46/54	0.1089325
Rapla	44/56	48/52	-0.1373626
Saare	47/53	48/52	-0.03628447
Tartu	49/51	46/54	0.1089325
Valga	45/55	47/53	-0.06861063
Viljandi	54/46	47/53	0.2871206
Võru	52/48	48/52	0.1602564

### 4.2 Micro-analysis

This section focuses on understanding gender interaction in the CDR data by exploring various users’ characteristics, including *gender*, *age*, *language*, and *location*. We also calculated gender segregation using the Coleman homophily index (*HI*) by considering the mentioned characteristics. In Section 4.2.1, we describe the *HI* in detail. The gender segregation based on the mentioned characteristics is explained from Section 4.2.2 to 4.2.5. We then extracted the relationships between the mentioned characteristics and compared them with census datasets from *Statistics Estonia* (https://www.stat.ee/en) in Section 4.2.6.

#### 4.2.1 Homophily index for measuring the segregation

Homophily is the tendency of individuals to connect and bond with other individuals [38]. In the past, homophily has been studied in great detail in various works [39–42]. These studies establish that similarity is associated with the connection among individuals and can be categorized based on age [43], gender [44], class [45], ethnicity [46], etc. In this work, we use the Coleman homophily index (*HI*) [47] to measure gender segregation in Estonia. We use *HI* as it efficiently compares the homophily of groups with different sizes by normalizing the excess homophily of groups by its maximal value [47].

*Calculating HI value*: Let us consider a network with static attribute groups *A* and *B* (of relative size *N*_*A*_ and *N*_*B*_ with *N*_*A*_ + *N*_*B*_ = 1) distributed among nodes uniformly at random and independently of the network structure, such that there is a fraction *P*_*AB*_ = *P*_*BA*_ of edges between groups, and fractions *P*_*AA*_, *P*_*BB*_ within each group (*P*_*AA*_ + *P*_*AB*_ + *P*_*BB*_ = 1). In the case of two attribute groups, the probability that a random edge from a node in a group *A* leads to a node in group *A* is defined as:

$$\begin{array}{c} T_{aa}=\frac{2P_{aa}}{2P_{aa}+P_{ab}} \end{array}$$ (1)

Similarly, we can write equation for *T*_*bb*_. The *HI* value for group *A* (*HI*_*A*_) and *B* (*HI*_*B*_) can be calculated using

$$\begin{array}{c} HI_{A}=\frac{T_{AA}-N_{A}}{1-N_{A}} \end{array}$$ (2)

$$\begin{array}{c} HI_{B}=\frac{T_{BB}-N_{B}}{1-N_{B}} \end{array}$$ (3)

The range for both *HI*_*A*_ and *HI*_*B*_ is from -1 to 1, where -1 for *HI*_*A*_ means that group *A* individuals only connects with group *B* individuals (only in between groups connections), whereas 1 for *HI*_*A*_ means that group *A* individuals only connects with group *A* individuals (only within-group connections). Similar is true for group *B* homophily index *HI*_*B*_.

#### 4.2.2 Gender segregation based on age-groups

**Fig 4:**
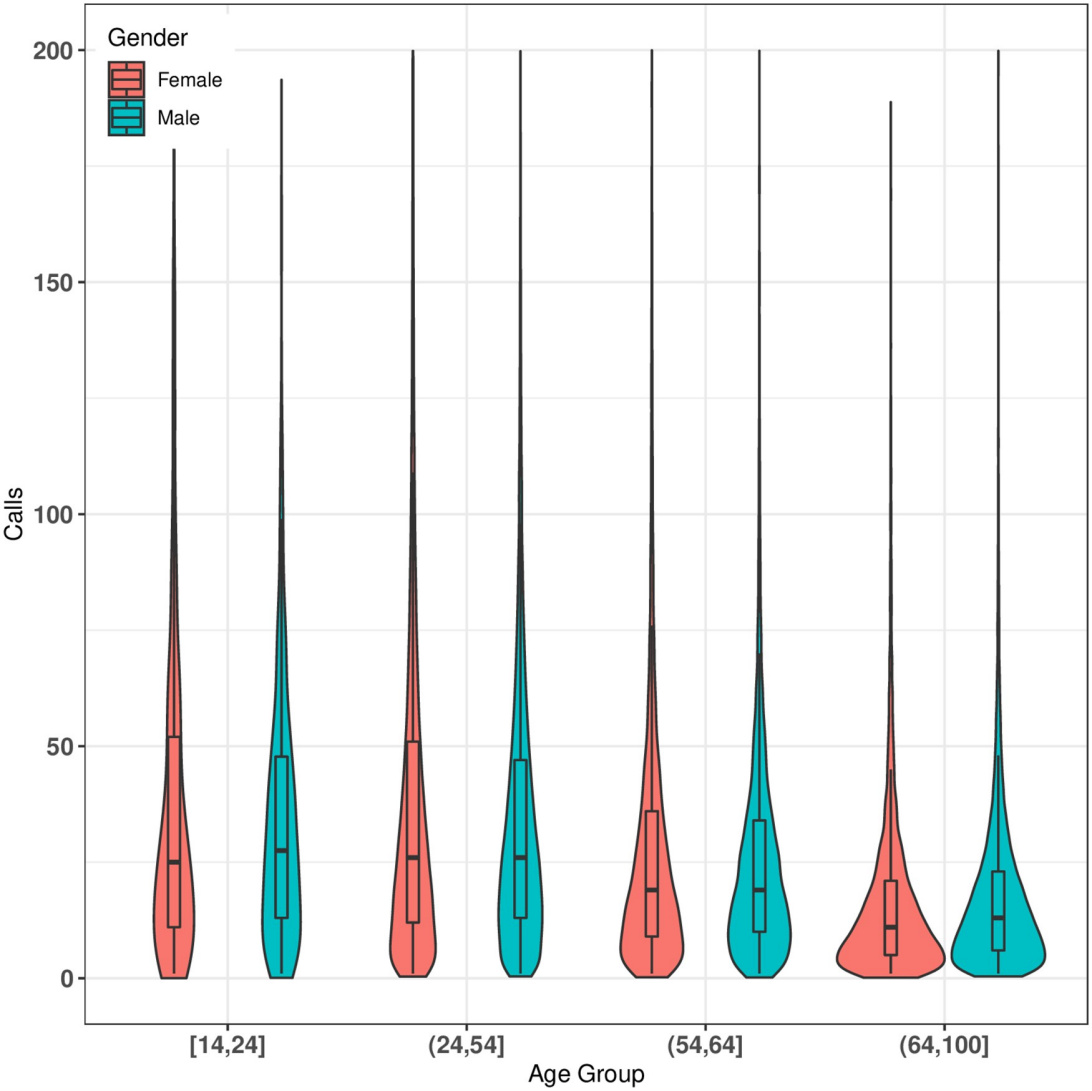
Median of the calls for various age-groups based on gender. Median of the calls for female’s age-groups (14,24], (24,54], (54,64] and (64,100] are 28, 27, 19 and 12 respectively. Similarly, median of the calls for male’s age-groups are 25, 27, 19 and 11 respectively.

In this section, we report gender segregation among users considering four different age-groups, that is, *early working age* ([14-24] years), *prime working age* ((24-54] years), *mature working age* ((54-64] years), and *elderly* (65+ years). Please note that we use the age-groups name and age-groups range interchangeably in the rest of the section. Fig 4 compares the calls made by various age-groups based on gender. We find that the difference between median of the females and median of the males calls is highest for the age-group [14, 24]. The median of the calls for females and males of age-group [14, 24] are 28 and 25, respectively. This indicates that females of age-group [14, 24] call more frequently than males. Next, we study gender segregation among various age-groups using *HI* values.

**Fig 5:**
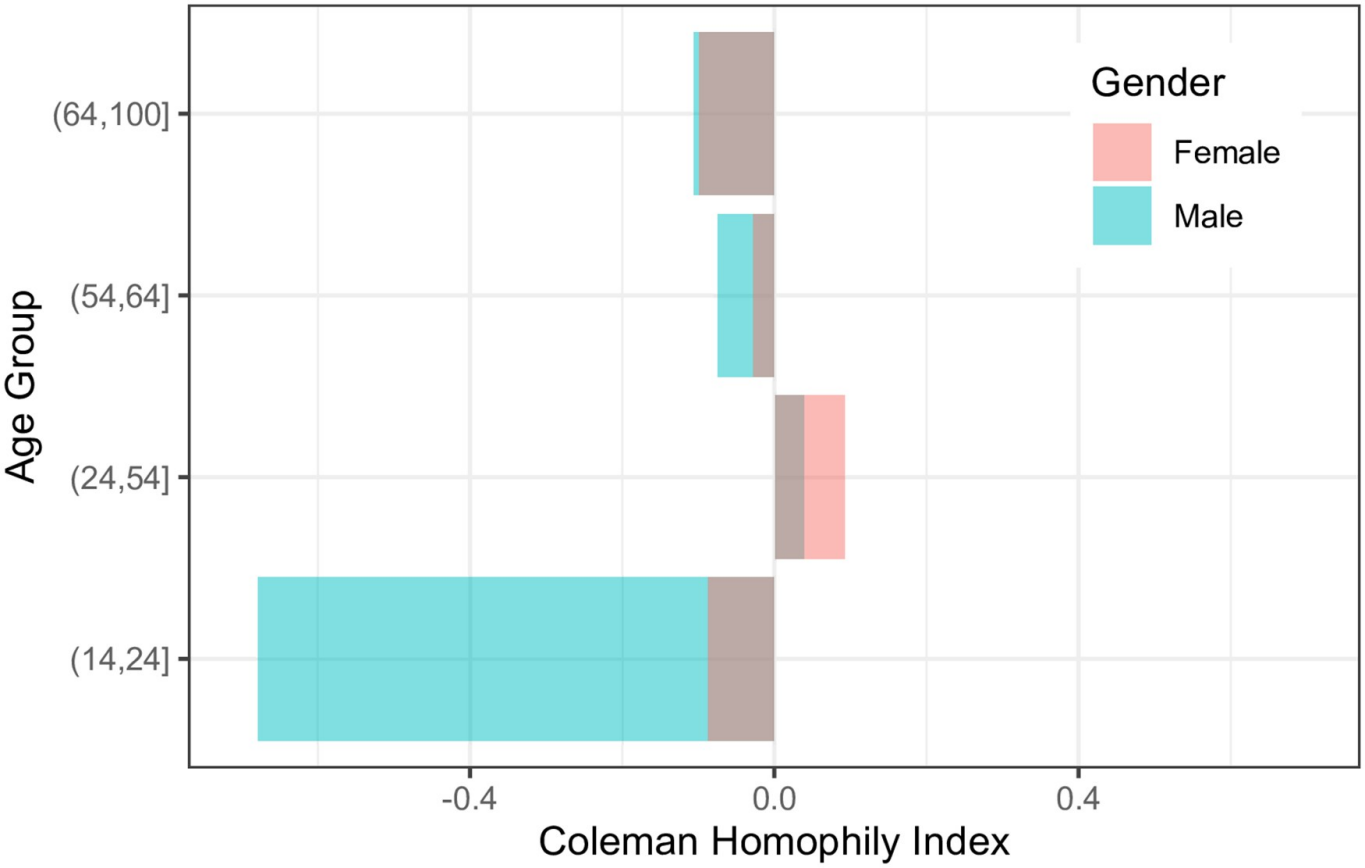
Coleman’s homophily index (*HI*) for various age-group. *HI* for female’s age-groups (14,24], (24,54], (54,64] and (64,100] are -0.09, 0.09, -0.03 and -0.1 respectively. Similarly, *HI* for male’s age-groups are -0.68, 0.04, -0.08 and -0.11 respectively.

Based on *HI* values (see Fig 5), we can infer that males and females in all the age-groups (except (24,54]) tend to call more to the opposite gender of the same age-group. Whereas calls of both females and males of age-group (24,54] are more likely to remain within the same gender and age-group. Also, males of age-group (14,24] are strongly inclined towards females of the same age-groups with *HI* value equals to -0.68. We can also conclude that females of age-group (54,64] and males of age-group (24,54] are well connected with both genders with low *HI* values 0.04 and -0.028, respectively. Age-group (64,100] exhibit similar connectivity behavior with the opposite gender within the same age-group with *HI* values of -0.11 and -0.1, respectively.

The comparison of females and males calling pattern based on age-groups also highlights that males of *early working age*, *mature working age*, and *elderly* calls more to females of the same age-group. Still, at the same time, they maintain strong connectivity with males of other age-groups as well. On the other hand, most females’ calls of *prime working age* remain within the same age-group of females, making their connectivity with other age-groups relatively weak. Based on these calling behavior between age-groups, we can conclude that the inclination of both females and males towards the same gender came from the *prime working age*. To explore further, next, we examined language-based gender segregation.

#### 4.2.3 Gender segregation based on language

**Fig 6:**
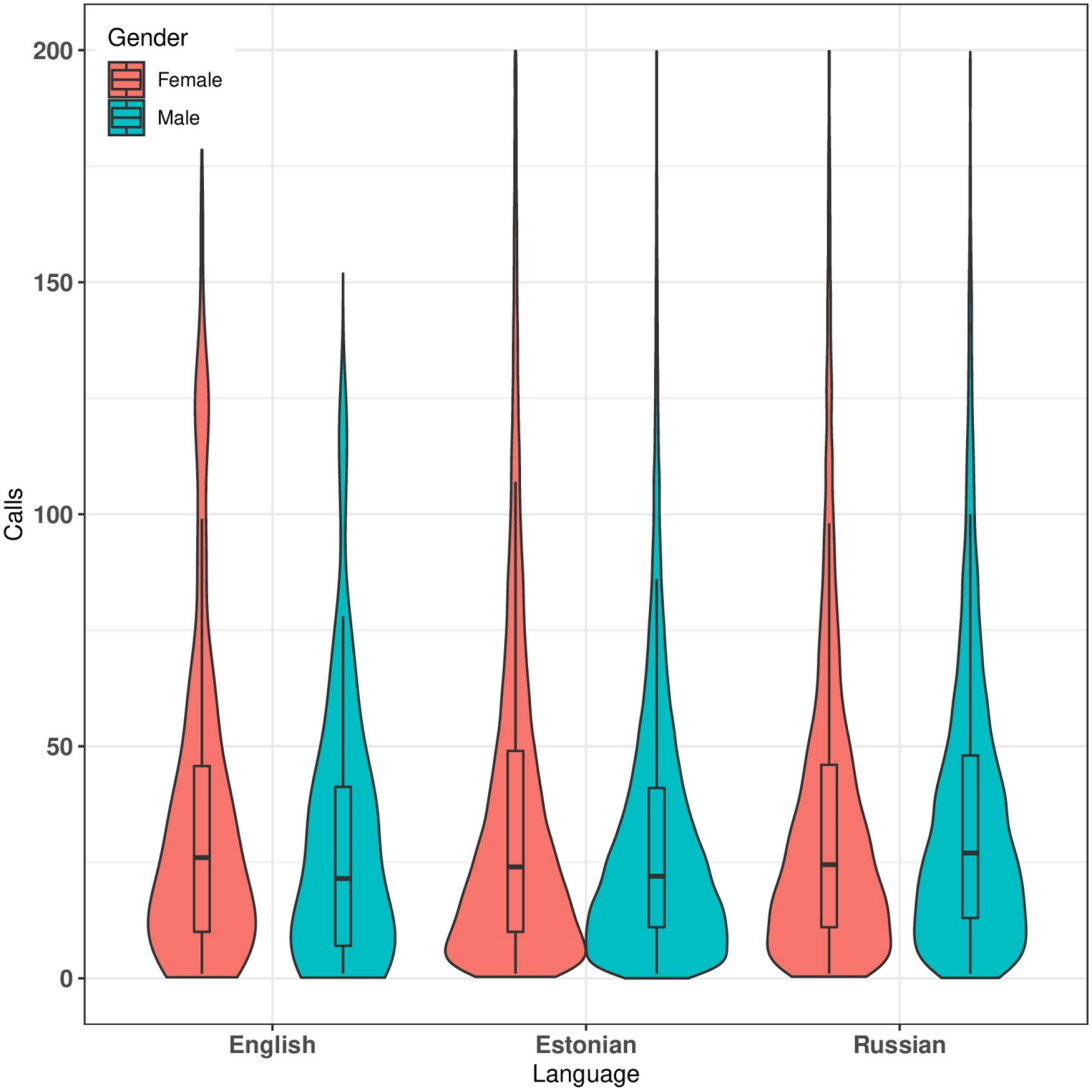
Median of the calls for various language speaking population based on gender. Median of the calls for female’s for languages English, Estonian and Russian are 26, 24 and 25 respectively. Similarly, median of the calls for male’s for languages are 21.5, 22 and 27 respectively.

As mentioned earlier, our dataset includes three languages spoken by the population of Estonia, namely *Estonian*, *Russian* and *English*. In this section, we begin our analysis by comparing the median of the calls for both females and males based on language (see Fig 6). Our findings highlight that Estonian-speaking females call more compare to Estonian-speaking males. On the other hand, Russian-speaking males call more compare to Russian-speaking females. We also observe that median of the calls made by Russian-speaking individuals (both males and females) and English-speaking males are higher than the Estonian-speaking population (both males and females). Based on call activity among different languages, we can infer that Russian-speaking individuals call comparatively higher than Estonian-speaking individuals.

**Fig 7:**
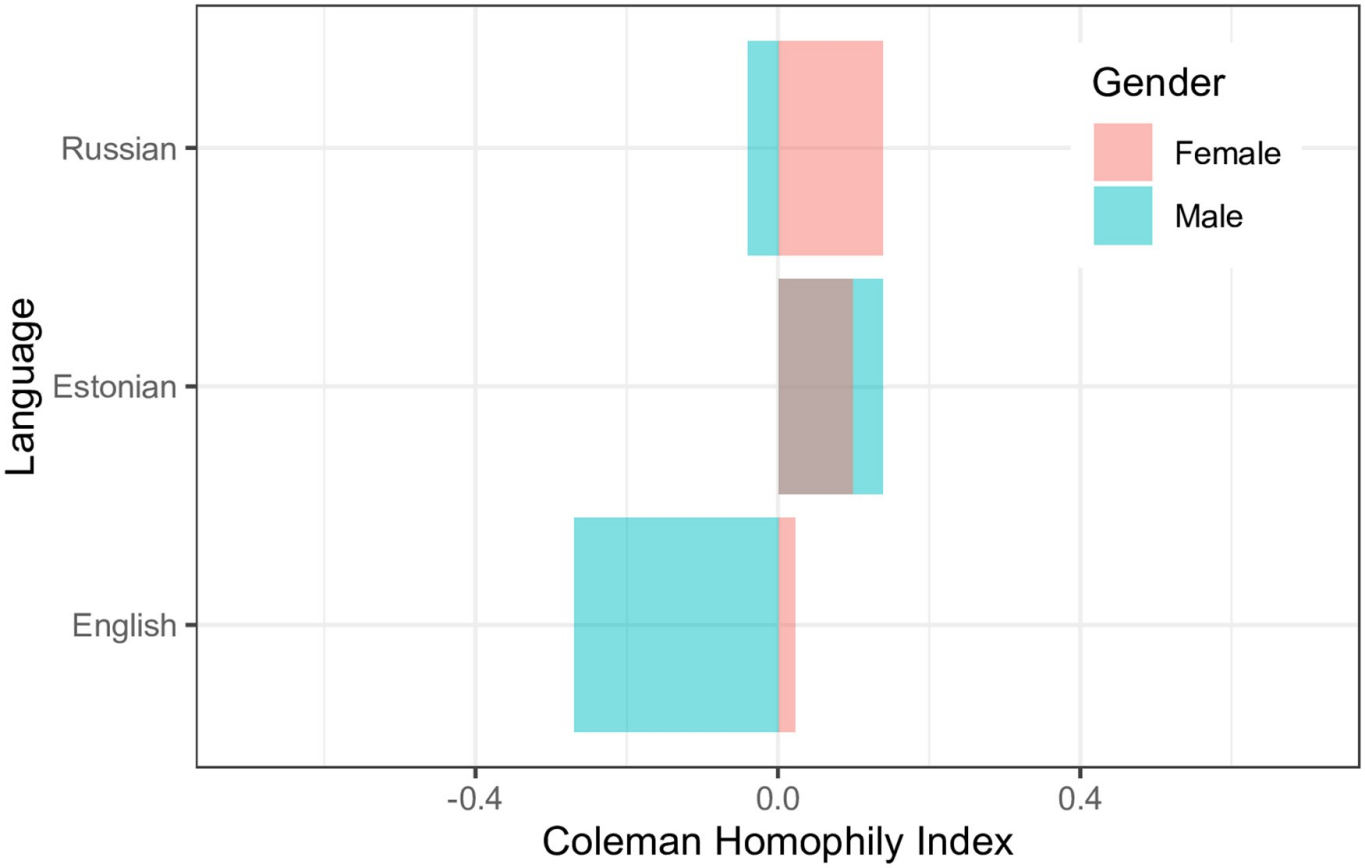
Coleman’s homophily index (*HI*) for various languages. *HI* for male’s languages English, Estonian and Russian are -0.27, 0.14 and -0.04 respectively. Similarly, *HI* for female’s age-groups are 0.02, 0.1 and 0.14 respectively.

Next, we measure gender segregation based on language using *HI* values. The *HI* index for Russian-speaking population shows that Russian-speaking females are more inclined towards Russian-speaking females (see Fig 7). On the other hand, Russian-speaking males are slightly inclined towards Russian-speaking females. The *HI* index for the Estonian-speaking population shows that both males and females are inclined towards the same gender and language. For the English-speaking population, the *HI* index indicates that both males and females like to talk more with females. Therefore, based on language, we can conclude that females are inclined towards the same language females. On the other hand, Russian and English-speaking males are inclined towards the same language females, but Estonian males are inclined towards Estonian-speaking males. This shows that Estonian-speaking population and Russian-speaking females are more segregated compared to others. To explore further, we also investigate gender segregation based on counties.

#### 4.2.4 Gender segregation in Estonian counties

**Fig 8:**
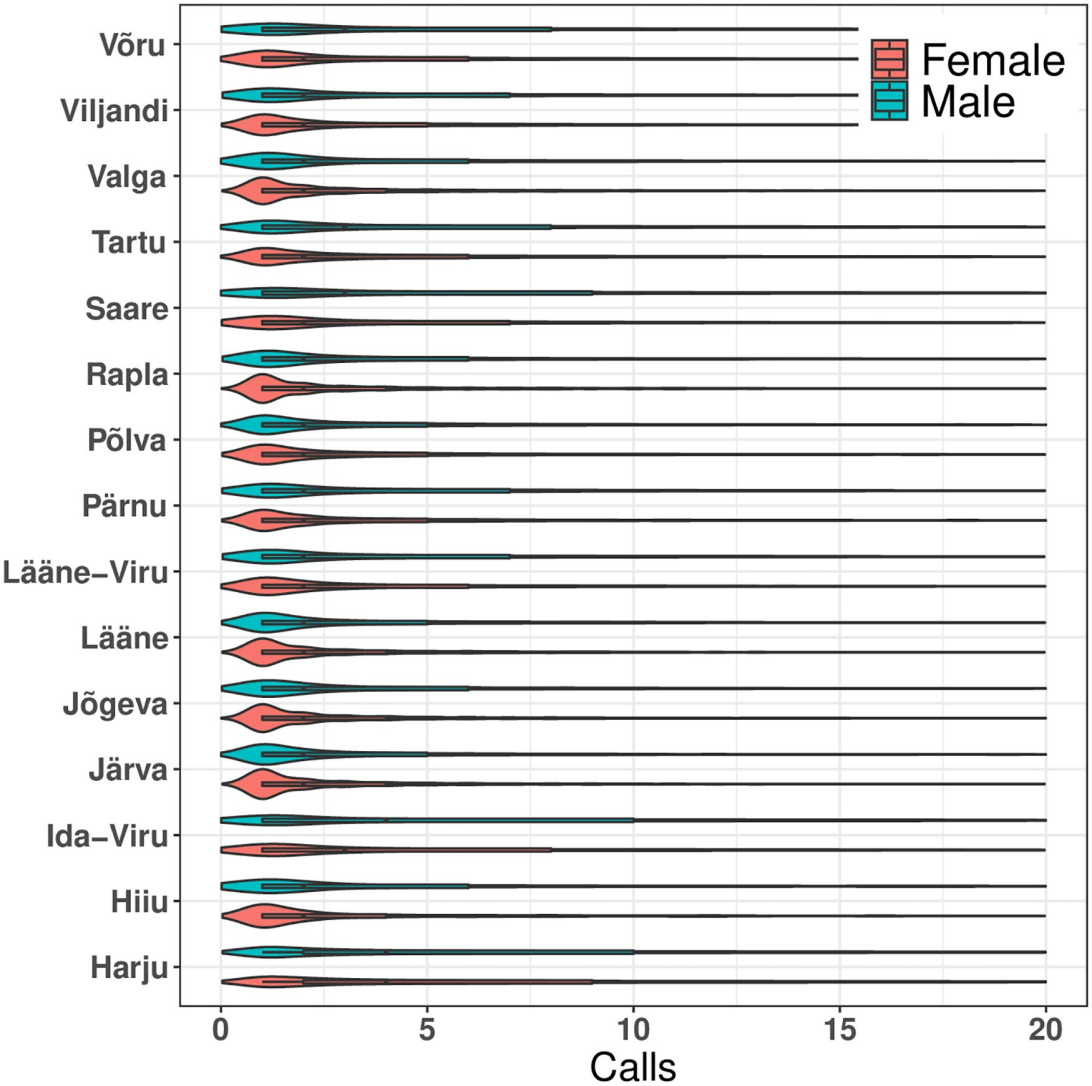
Calls density for counties in Estonia based on gender. Median of the calls for male’s in various counties (starting from bottom (Harju) to top (Võru)) are 7, 2, 4, 2, 2, 2, 5, 2, 2, 2, 3, 3, 2, 2 and 3 respectively. Similarly, median of the calls for female’s in various counties are 7, 4, 7, 2, 2, 2, 3, 3, 2, 2, 5, 4, 2, 3 and 4 respectively.

There are 15 counties in Estonia, with Harju county, which includes capital Tallinn being the most populous and Hiiu county being the least populous. Here, we start our analysis by comparing the median of the calls made in various counties based on gender, as shown in Fig 8. We find that in total, 8 counties (i.e., Hiiu, Ida-Viru, Lääne-Viru, Pärnu, Saare, Tartu, Viljandi, and Võru) have a difference in calls by gender. We also observe that even though the distribution of the gender population in the *Harju* and *Ida-Viru* counties are the same (see Table 4), county *Harju* has the least gap in terms of males and females median of the calls; and the county *Ida-Viru* has the biggest gap. In reality, there is around 81% Russian-speaking and 18% Estonian-speaking population in *Ida-Viru*. On the other hand, *Harju* has approximately 40% Russian-speaking and 60% Estonian-speaking population. This indicates that the Russian-speaking females tend to call more compared to the Estonian-speaking population. We investigate this in more detail in the next section.

**Fig 9:**
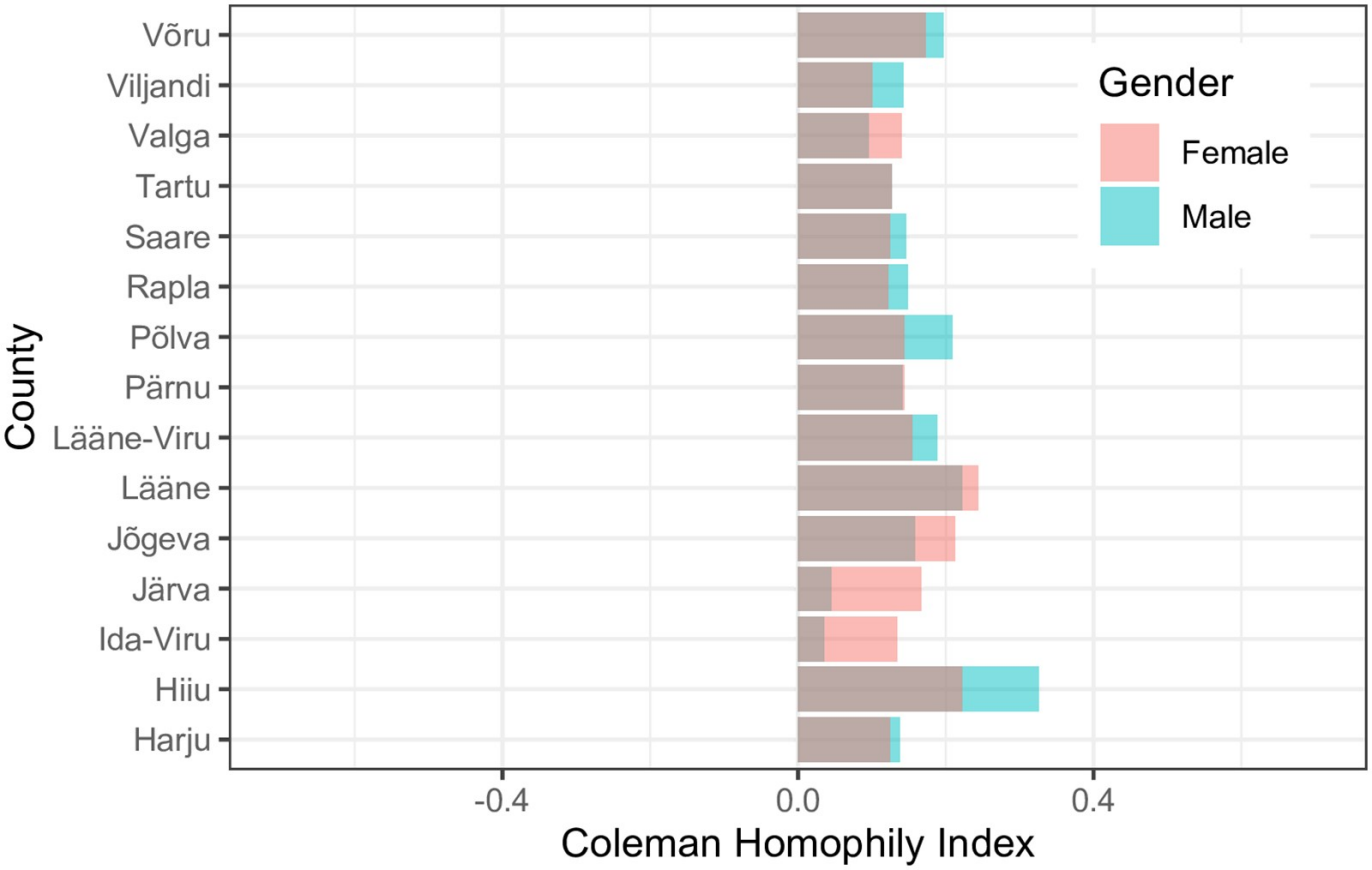
Coleman’s homophily index (*HI*) for various counties. *HI* for male’s in various counties (starting from bottom (Harju) to top (Võru)) are -0.27, 0.14 and -0.04 respectively. Similarly, *HI* for female’s age-groups are 0.02, 0.1 and 0.14 respectively.

Additionally, to understand gender segregation in the Estonian counties, we calculated the *HI* values for both males and females separately in each county. We observe that in all counties, both males and females are inclined towards the same gender (see Fig 9). In counties (Hiiu, Lääne, Põlva, etc.), males are more inclined towards other males. Similarly, females are more inclined towards other females in Lääne, Hiiu, Jõgeva, etc. We further find that the counties with the most differences between *HI* values for males and females are Järva (0.12), Hiiu (0.11), Ida-Viru (0.11), and Põlva (0.07). Also, the counties with the least difference between *HI* value for males and females are Tartu (0.0002), Pärnu (0.002), and Harju (0.01). In the next section, we study two Estonian counties: Harju and Ida-Viru, to further explore gender segregation.

#### 4.2.5 Case study of prime working age individuals in Harju & Ida-Viru

In this section, we aim to compare gender segregation based on languages specifically in Harju and Ida-Viru counties. We focus on these counties for the following reasons. First, although the actual population of Harju is 6 times greater than Ida-Viru, however, the percentage of males and females in Harju and Ida-Viru is same, that is, 45% males and 55% females. Second, in Harju majority population is Estonian-speaking, and in Ida-Viru, the majority population is Russian-speaking. In particular, Harju has 40% Russian-speaking and 60% Estonian-speaking population, and on the other hand, Ida-Viru has roughly 81% Russian-speaking and 18% Estonian-speaking population.

Additionally, we focus on *prime working age* population (i.e., age-group (24,54]) only. This is because the overall prime working age population is more inclined toward the same gender and at the same time, it covers more than 56% of our users’ dataset. Furthermore, we filter our data for the Estonian and Russian-speaking population only as it covers more than 99% of individuals in our dataset. After applying the age-group, language, and location filters, we are still covering more than 13.5% of our dataset.

Table 5 covers various possible cases on filtered dataset (Row # 1 to 6) based on (i) languages, and (ii) location. We also report three additional cases (Table 5, Row # 7 to 9) for comparison with previous cases on filtered dataset. Based on our analysis, we can conclude that

1. Prime working age Estonian-speaking population (both females and males) are more inclined towards same age-group, language and gender individuals (see Table 5, Row # 3, 4 and 8).
2. Prime working age Russian-speaking both males and females are more inclined towards prime working age Russian-speaking females (Table 5, Row # 5, 6 and 9).
3. In Harju, we can notice that Russian-speaking prime working age females are more segregated compared to Estonian-speaking prime working age females (Table 5, Row # 1, 3 and 5).
4. Similarly, in Ida-Viru, both Estonian-speaking and Russian-speaking prime working age females are equally segregated (Table 5, Row # 2, 4 and 6). On the other hand, Estonian-speaking prime working age males are more segregated than Russian-speaking prime working age males.

**Table 5:** Coleman’s homophily index (*HI*) for Case Study of prime working age population.

Row #	Case	*HI*_*females*_	*HI*_*males*_	Interpretation
1	Estonian & Russian-speaking prime working age population in Harju county	0.1	0.02	Females are more segregated
2	Estonian & Russian-speaking prime working age population in Ida-Viru county	0.13	-0.07	Females are more segregated
3	Estonian-speaking prime working age population in Harju county	0.05	0.05	Both females and males are equally segregated
4	Estonian-speaking prime working age population in Ida-Viru county	0.13	0.13	Both females and males are equally segregated
5	Russian-speaking prime working age population in Harju county	0.11	-0.03	Females are more segregated
6	Russian-speaking prime working age population in Ida-Viru county	0.12	-0.14	Both females and males prefer to connect with females
7	Estonian & Russian-speaking prime working age population in all counties	0.09	0.01	Females are more segregated
8	Estonian-speaking prime working age population in all counties	0.09	0.03	Females are more segregated
9	Russian-speaking prime working age population in all counties	0.12	-0.12	Both females and males prefer to connect with females

#### 4.2.6 Validating CDR using Estonian census data

Next, we compare the distribution of gender, age-groups, language, and locations from CDR data with publicly available Estonian census datasets. The reason for doing this is to understand the representation of different users’ groups in the CDR dataset relative to the actual population. We failed to find out a dataset that covers all users’ features present in the CDR dataset (i.e., *gender*, *age*, *language* and *location*). At last, we came across two separate publicly available datasets that cover three features each. The first dataset (http://pub.stat.ee) includes the *gender, age* and *location* of the Estonian population; while *gender*, *language* and *location* are present in the second dataset (http://andmebaas.stat.ee). Both these datasets are publicly available on the website of Statistics Estonia. Thus, we decide to use these two datasets to compare the representation of Estonian population in CDR dataset. On comparing, we find that CDR covers different percentages of actual age-groups and language population. For example, CDR data covers 18.1% of actual female prime working age population (see Table 6, Row 2). Similar comparison findings are also reported based on language (see Table 7). Thus, we argue that CDR can provide an opportunity to extract meaningful relationships among users’ features.

**Table 6:** The representation of various gender and age-group in the CDR data in comparison to the census data.

Gender	Age-Group	CDR/Actual Pop (%)
Female	(14,24]	1.1
Female	(24,54]	18.1
Female	(54,64]	15
Female	(64,100]	6
Male	(14,24]	1.1
Male	(24,54]	18
Male	(54,64]	17.3
Male	(64,100]	8.6

**Table 7:** The representation of various gender and language in the CDR data in comparison to the census data.

Gender	Language	CDR/Actual Pop (%)
Female	English	85.5
Female	Estonian	18.8
Female	Russian	5
Male	English	91.1
Male	Estonian	20
Male	Russian	5.8

**Fig 10:**
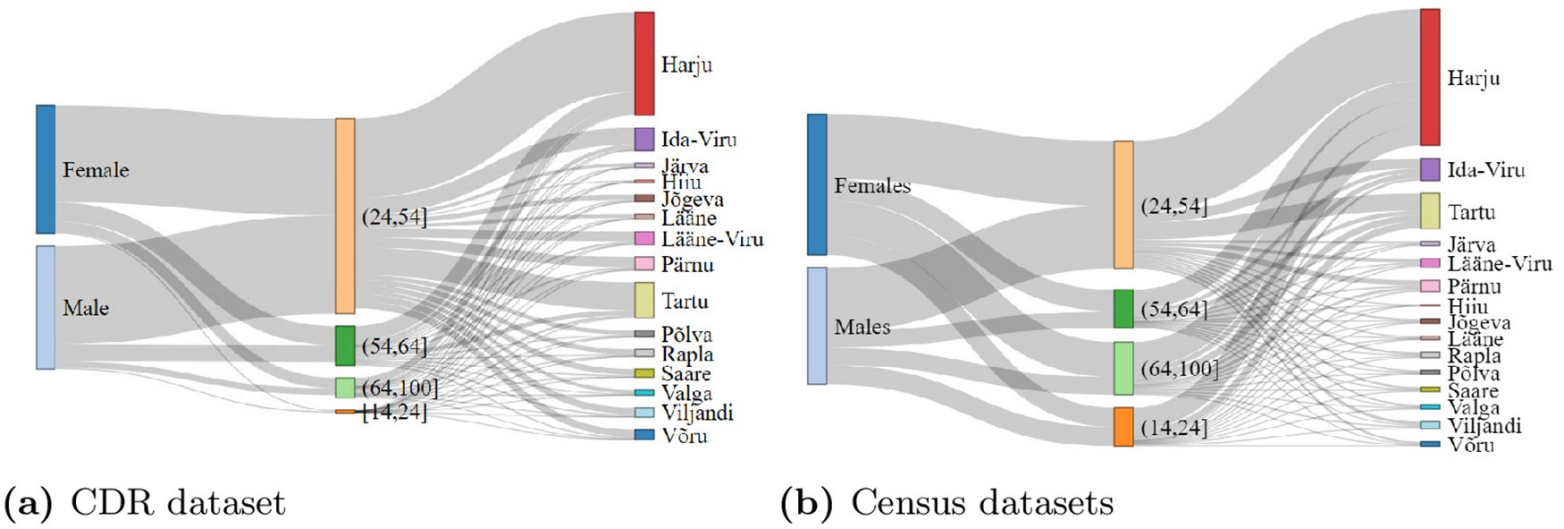
Comparison of gender, age-group, and county relation using (a) CDR records, and (b) census data from Statistics Estonia. In both figures (a) and (b), leftmost bars represent *gender*, middle bars represent *age-group*, and rightmost bars show the *counties of Estonia*.

From CDR data, we observe that mobiles are commonly used by prime working age users (i.e., (24,54]) and mature working age users (i.e., (54,64]). These two categories cover 56.37% and 22.94% of our dataset, respectively (see Fig 10). Mobile usage percentages for early working age and elderly users are 0.16% and 20.53%, respectively. On the other hand, the actual population percentage under prime working, mature working, early working, and elderly age-groups are 49.63%, 14.82%, 15%, and 20.55%, respectively. From this, we can infer that in the CDR dataset, the representation of the prime working age users is significant considering their actual population in Estonia. Thus, we can argue that the prime working age users’ findings can be considered accurate with reasonable confidence. The same is true for mature working age, and elderly users. On the other hand, the representation of early working age-group in CDR is less to negligible than the actual population, making it difficult to make any concrete statements about this age-group. This distribution is further compared in counties for both actual and CDR data. We find that distributions for early working again lack similarity with actual population, as we observed earlier when comparing age-group’s distribution for CDR with the actual data.

**Fig 11:**
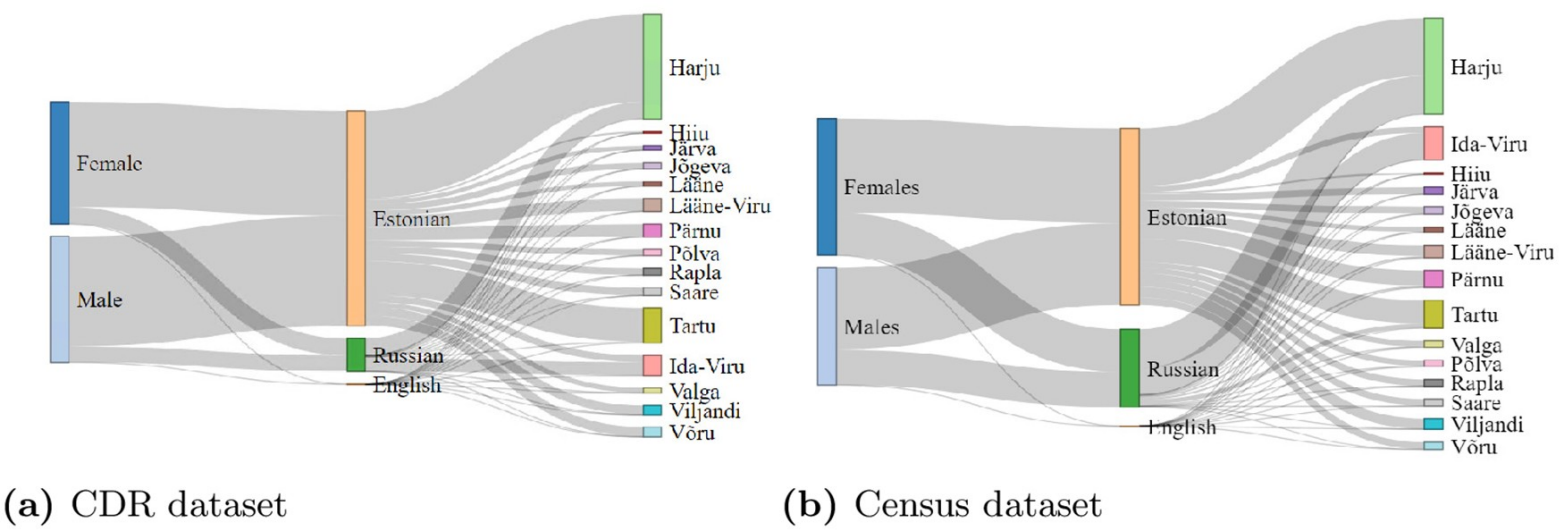
Comparison of gender, language and county relation using (a) CDR records, and (b) census data from Statistics Estonia. In both figures (a) and (b), leftmost bars represent *gender*, middle bars represent *language*, and rightmost bars show the *counties of Estonia*.

Similarly, we also compare the language information present in CDR dataset for gender and counties (see Fig 11(a)) with census datasets (see Fig 11(b)). The representation of Estonian, Russian and English-speaking users in CDR is 87.09%, 12.7%, and 0.21%; and in actual population data is 69.38%, 30.6% and 0.02%. We can conclude that representation of Estonian-speaking population in CDR data is 1.25 times higher than that of the actual population. On the other hand, CDR representation of Russian-speaking population is 2.41 times smaller compared to their actual population in Estonia. Since the representation of Estonian and Russian-speaking population covers more than 99% in reality and also in CDR data, the results of this study using CDR data can be considered useful for understanding gender segregation based on Estonian and Russian language in Estonia.

**Fig 12:**
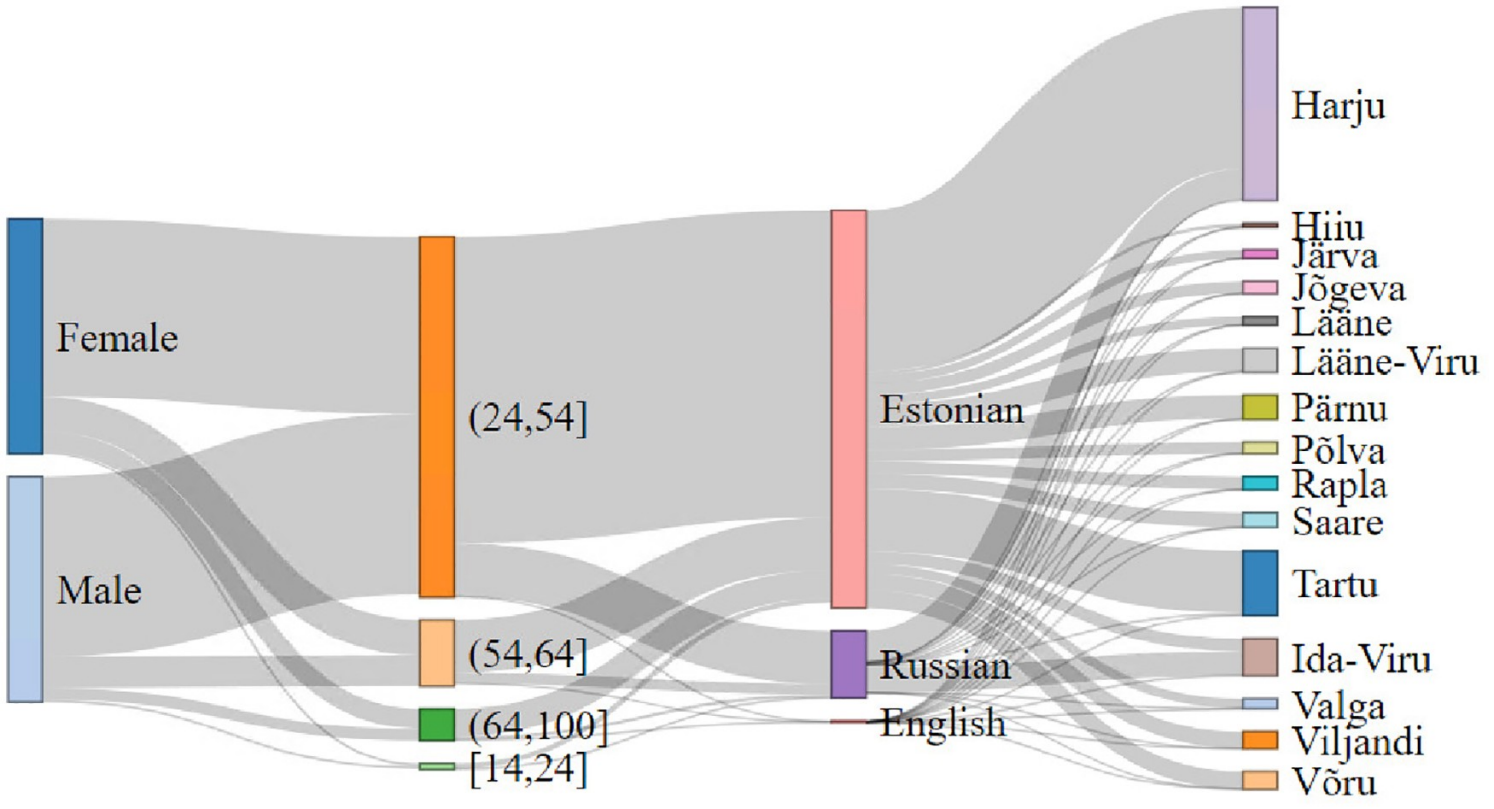
Comparison of gender, age-group, language, and county relation using CDR data. Here, leftmost bars represent *gender*, second leftmost bars represent *age-group*, second rightmost bars shows *language*, and rightmost bars represent the *counties of Estonia*.

We note that the CDR dataset and actual dataset can vary at various levels, such as: (1) the percentage of prime working age representation is higher in CDR compared to the actual population in Estonia, which is further noticed in the distribution of these age-groups by counties. (2) The nature of the data itself reduces the representation of early working age and old age users, which we confirm by comparing CDR and actual data. (3) We also observe that the CDR data has a higher representation of Estonian-speaking users and lower Russian-speaking users than the actual population, which can be seen as another limitation of the CDR dataset. (4) Finally, we can deduce the distribution for gender, age-group, language, and county using CDR data, which resembles the reality of Estonian society with the above-mentioned limitations (see Fig 12).

## 5 Conclusion

Understanding gender segregation and integration play a critical role in determining any nation’s social and economic development of any nation [48]. With a quest to understand gender segregation in Estonia, we analyze a large call data records provided by one of the biggest mobile operators in Estonia. We analyze the data from the following two broad dimensions:

1. Firstly, we perform the *macro-analysis* for understanding gender segregation using social network analysis. The results on network analysis indicate that most females and males call within the same gender, and the female network is relatively spread out more compared to the males’ network, which is dense and strongly connected. In particular, to understand gender segregation in terms of identifying the top social connectors, we perform the analysis at the county level as well, and we observed that in all counties, both males and females are inclined towards the same gender.
2. Secondly, we study the impact of various users’ features to explore the segregation in detail (*micro-analysis*). In particular, we analyze the impact of age, language, and location on gender segregation. We find that the prime working age population (i.e., (24,54] years) is more segregated compared to other age-groups. Furthermore, we find that the Estonian-speaking population (both males and females) tend to communicate more with the Estonian-speaking population and same gender.

We compare the relationships among features of the CDR dataset with the Estonian census data, and find that we can deduce the substantial relationships between users’ characteristics such as gender, age, language, location using the CDR dataset. These relationships can be used by government agencies to make target policies for the needed segregated group in particular. We further noticed that the major limitation of the CDR dataset comes from the fact that mobile phone use is widespread after a certain age, therefore, CDR data can be considered as a valuable guide for understanding the communication pattern in adults.

We plan to include various future directions for this work. To grasp the economic inequality, we intend to analyze the data to a more detailed location, such as the municipalities. We would like to investigate a larger dataset that spans a longer period of time. We would also like to identify potential factors that are responsible for gender segregation in society. We would also like to combine the mobile CDR data with other datasets such as *financial* data to understand the socioeconomic segregation in Estonia.
